# Performance of the IOTA ADNEX model combined with HE4 for identifying early-stage ovarian cancer

**DOI:** 10.3389/fonc.2022.949766

**Published:** 2022-09-16

**Authors:** Suying Yang, Jing Tang, Yue Rong, Min Wang, Jun Long, Cheng Chen, Cong Wang

**Affiliations:** ^1^ Department of Ultrasonography, Chongqing Health Center for Women and Children, Chongqing, China; ^2^ Department of Ultrasonography, Women and Children’s Hospital of Chongqing Medical University, Chongqing, China

**Keywords:** ovarian cancer (OC), IOTA ADNEX model, human epididymis protein 4 (HE4), serum cancer antigen-125 (CA 125), receiver-operating characteristics (ROC) curve

## Abstract

**Objective:**

This work was designed to investigate the performance of the International Ovarian Tumor Analysis (IOTA) ADNEX (Assessment of Different NEoplasias in the adneXa) model combined with human epithelial protein 4 (HE4) for early ovarian cancer (OC) detection.

**Methods:**

A total of 376 women who were hospitalized and operated on in Women and Children’s Hospital of Chongqing Medical University were selected. Ultrasonographic images, cancer antigen-125 (CA 125) levels, and HE4 levels were obtained. All cases were analyzed and the histopathological diagnosis serves as the reference standard. Based on the IOTA ADNEX model post-processing software, the risk prediction value was calculated. We analyzed receiver operating characteristic curves to determine whether the IOTA ADNEX model alone or combined with HE4 provided better diagnostic accuracy.

**Results:**

The area under the curve (AUC) of the ADNEX model alone or combined with HE4 in predicting benign and malignant ovarian tumors was 0.914 (95% CI, 0.881–0.941) and 0.916 (95% CI, 0.883–0.942), respectively. With the cutoff risk of 10%, the ADNEX model had a sensitivity of 0.93 (95% CI, 0.87–0.97) and a specificity of 0.73 (95% CI, 0.67–0.78), while combined with HE4, it had a sensitivity of 0.90 (95% CI, 0.84–0.95) and a specificity of 0.81 (95% CI, 0.76–0.86). The IOTA ADNEX model combined with HE4 was better at improving the accuracy of the differential diagnosis between different OCs than the IOTA ADNEX model alone. A significant difference was found in separating borderline masses from Stage II–IV OC (*p* = 0.0257).

**Conclusions:**

A combination of the IOTA ADNEX model and HE4 can improve the specificity of diagnosis of ovarian benign and malignant tumors and increase the sensitivity and effectiveness of the differential diagnosis of Stage II–IV OC and borderline tumors.

## Introduction

Ovarian cancer (OC), one type of gynecologic malignancy with a high mortality rate, has seriously threatened the life and health of women, whose incidence and mortality rate are gradually increasing over the years (1–4). The 5-year survival rate of advanced OC is about 30%, according to past reports. In contrast, postoperative survival rates of early-stage OC can reach 92.6%, while early diagnosis accuracy is just 16.3% (5). To enhance the accuracy of ovarian tumor ultrasound diagnosis, a number of prediction models have been developed by the International Ovarian Tumor Analysis (IOTA) group utilizing logistic regression analysis, which include the LR1, LR2, and IOTA ADNEX (Assessment of Different NEoplasias in the adneXa) models (6–8). As per related studies, the IOTA ADNEX model is the most effective in differentiating benign from malignant ovarian tumors.

Performing a suitable first surgical procedure is crucial for OC patients, which depends on the correct staging of the tumor before surgery. The IOTA group delivered the ADNEX model in 2014, which was the first to distinguish between benign ovarian tumors, borderline, invasive, and secondary metastatic cancers. An explanation of how to apply the ADNEX model from the IOTA group for discriminating between different subtypes of adnexal tumors was provided by Van Calster et al. (9). Related research shows that the model discriminated well between benign tumors and each of the four types of malignancy, with AUCs ranging between 0.85 and 0.99. Nevertheless, an ovarian borderline, a Stage I OC, or a metastatic ovarian tumor cannot be accurately differentiated with it (9–14). In addition, with regard to the IOTA ADNEX cutoff risk, the guidelines merely recommended the selection according to the type of center and the clinical characteristics of the patient, without an accurate value. A cutoff risk of 10% was mostly recommended in research, which has a high sensitivity (>90%) despite its low specificity (approximately 62%) (15, 16).

Detecting ovarian epithelial cancer at an earlier stage may be possible by combining tumor markers (17). Human epithelial protein 4 (HE4) is a highly recognized clinical marker for epithelial ovarian tumors after CA125 and usually applied in combination with CA125 to determine the benignity and malignancy of ovarian tumors. Specifically, it is superior to CA125 in detecting borderline and early-stage OC, and has been approved for evaluating follow-up and recurrence of OC patients (18–23).

At present, there is a lack of reports on the combined diagnosis of the IOTA ADNEX model and HE4. Therefore, this work proposed to combine the IOTA ADNEX model containing CA125 with HE4 to analyze its diagnostic efficacy and provide a reference for OC early detection.

## Methods and materials

### Setting of study and patients

An evaluation of diagnostic accuracy was conducted retrospectively in one hospital, a tertiary referral oncology center located in Chongqing, China, the Women and Children’s Hospital of Chongqing Medical University. This study consecutively enrolled 405 women diagnosed with an adnexal mass *via* ultrasound from August 2017 to September 2020. The following were the inclusion criteria (1): patients were older than 14 years old (2); patients were all examined at the Women and Children’s Hospital of Chongqing Medical University before surgery, and the serum CA125 and HE4 levels, ultrasound image workstation and report data were complete; and (3) patients’ postoperative pathological diagnosis was definite. Exclusion criteria were patients with adnexal masses not derived from ovarian tissue. Ethics approval for research is provided by the Institutional Ethics Committee of Women and Children’s Hospital of Chongqing Medical University.

Most of these patients have abdominal masses, abdominal pain, abdominal distension, and vaginal bleeding, while others are found accidentally during physical examinations. The examination was performed by a gynecologic ultrasonographer at the Women and Children’s Hospital of Chongqing Medical University. Ultrasound machines used in the study were the GE Voluson E8 or E10 (GE Healthcare, Zipf, Austria), with transvaginal probes measuring 5.0–9.0 MHz and transabdominal probes measuring 2–7 MHz. For patients with no sexual history, transabdominal exploration was performed after filling the bladder, and transrectal ultrasonography was performed if necessary. Transvaginal ultrasonography was used for patients with a history of sexual intercourse. For larger tumors, a combination of transcavitary and transabdominal ultrasound is used. According to the IOTA group’s terminology and methods to evaluate the morphology of ultrasonographic tumors (24), if a patient has a number of adnexal masses, we choose the mass exhibiting the most complicated ultrasound morphology, and if masses are morphologically similar, the larger mass is used (15).

During the ultrasound examination, we collected the patient’s age, menopausal status, and chemiluminescence measurements (Abbott i2000 analyzer, USA) of CA125 and HE4 before surgery.

#### ADNEX model

Cell phone applications for the IOTA ADNEX model are available. There are six ultrasound variables as well as three clinical variables in the model: age (years), referral center for an oncology or a non-oncology center, serum CA125 level (U/ml), maximum lesion diameter (mm), lesion diameter at its largest solid component (mm), cyst locules exceeding 10 (yes/no), amount of papillary projections (0, 1, 2, 3, or >3), ascites, or acoustic shadows present (yes/no). After inputting all the predictors objectively, as a result of the model, an absolute risk (in percentage terms) estimate is generated for five types of lesions in the adnexa. Furthermore, a malignancy risk estimate that incorporates all subtypes of malignancy is presented.

#### Reference standard

Reference standard was the histological pathological diagnosis results of the surgical specimens. These samples were examined by pathologists of our hospital and the ultrasound results were unknown. Tumors were classified based on guidelines of the World Health Organization for the classification of tumors (25). Stages of malignant tumors were determined by the new International Federation of Gynecology and Obstetrics criteria (26). A final diagnosis identified the five types of masses: benign, a borderline ovarian tumor (BOT), a Stage I OC, a Stage II–IV OC, and an ovarian metastatic cancer (**Table 1**).

**Table 1:** Pathological types of ovarian tumors in 376 patients.

Tumor pathology	*n* (%)
**Benign**	**259 (68.9)**
Mucinous cystadenoma	87 (23.0)
Serous cystadenoma	64 (17.0)
Cystadenofibroma	6 (1.6)
Seromucinous cystadenoma	12 (3.2)
Parovarian cyst	3 (0.8)
Endometriosis cyst	6 (1.6)
Serous adenofibroma	13 (3.5)
Serous surface papilloma	2 (0.5)
Theca cell tumor	11 (2.9)
Teratoma	35 (9.3)
Brenner tumor	2 (0.5)
Fibroma	7 (1.9)
Corpus luteum hematoma	1 (0.3)
Other ovarian benign lesion	10 (2.7)
**Borderline ovarian tumor**	**62 (16.5)**
Mucinous	23 (6.1)
Serous	35 (9.3)
Serous micropapillary type	1 (0.3)
Seromucinous	3 (0.8)
**Primary ovarian malignant**	**55 (14.6)**
Mucinous adenocarcinoma	3 (0.8)
Serous high-grade carcinoma	13 (3.5)
Clear cell carcinoma	8 (2.1)
Immature teratoma	2 (0.5)
Dysgerminoma	2 (0.5)
Endometrioid adenocarcinoma	4 (1.1)
Large cell neuroendocrine Carcinoma	1 (0.3)
Ovarian gonadal sex cord stromal tumor	1 (0.3)
Keratinizing squamous cell carcinoma	1 (0.3)
Granulosa-cell tumor	1 (0.3)
Yolk sac tumor	1 (0.3)
Poorly differentiated carcinoma	2 (0.5)
Carcinosarcoma	1 (0.3)
Seromucinous carcinoma	5 (1.3)
Adult granulose cell tumor	2 (0.5)
Rare primary invasive pathologies	4 (1.1)
**Ovarian metastasis**	**4 (1.1)**

#### Statistical analysis

MedCalc Statistical Software version 19.4.1 (MedCalc Software Ltd, Ostend, Belgium; https://www.medcalc.org; 2020) and IBM SPSS Statistics for Windows, Version 20.0 (Armonk, NY: IBM Corp) were used for statistical analysis. For statistical purposes, a borderline tumor was categorized as malignant.

An analysis of the ADNEX model and its combination with HE4 is based on receiver operating characteristic (ROC) curves. The area under the curve (AUC) with 95% confidence intervals (CIs) was calculated and the total risk of malignancy was used to distinguish benign from malignant tumors. AUC values of the different subclassification of malignant tumors were also calculated for analysis. The cutoff risks of 5%, 10%, and 15% of the ADNEX model were selected as the total risk of malignancy (for instance, calculate the risk of four different malignancies as a sum) in separating benign from malignant ovarian tumors, and the sensitivity and specificity, as well as the predictive values and likelihood ratios, were calculated. Additionally, DeLong’s test was applied to compare the performance in identifying different subtypes of ovarian tumors when the ADNEX model was used alone or was combined with HE4.

In this work, tumor ultrasonographic characteristics, brief population statistics of patients, a description of the clinical features, and an analysis of tumor markers were conducted. If data are categorical, the chi-square test and Fisher’s exact test should be used, and if data are continuous, the Mann–Whitney *U* test should be used. All comparisons were statistically significant at *p* < 0.05.

## Results

### Pathologic diagnosis and clinical findings

**Figure 1:**
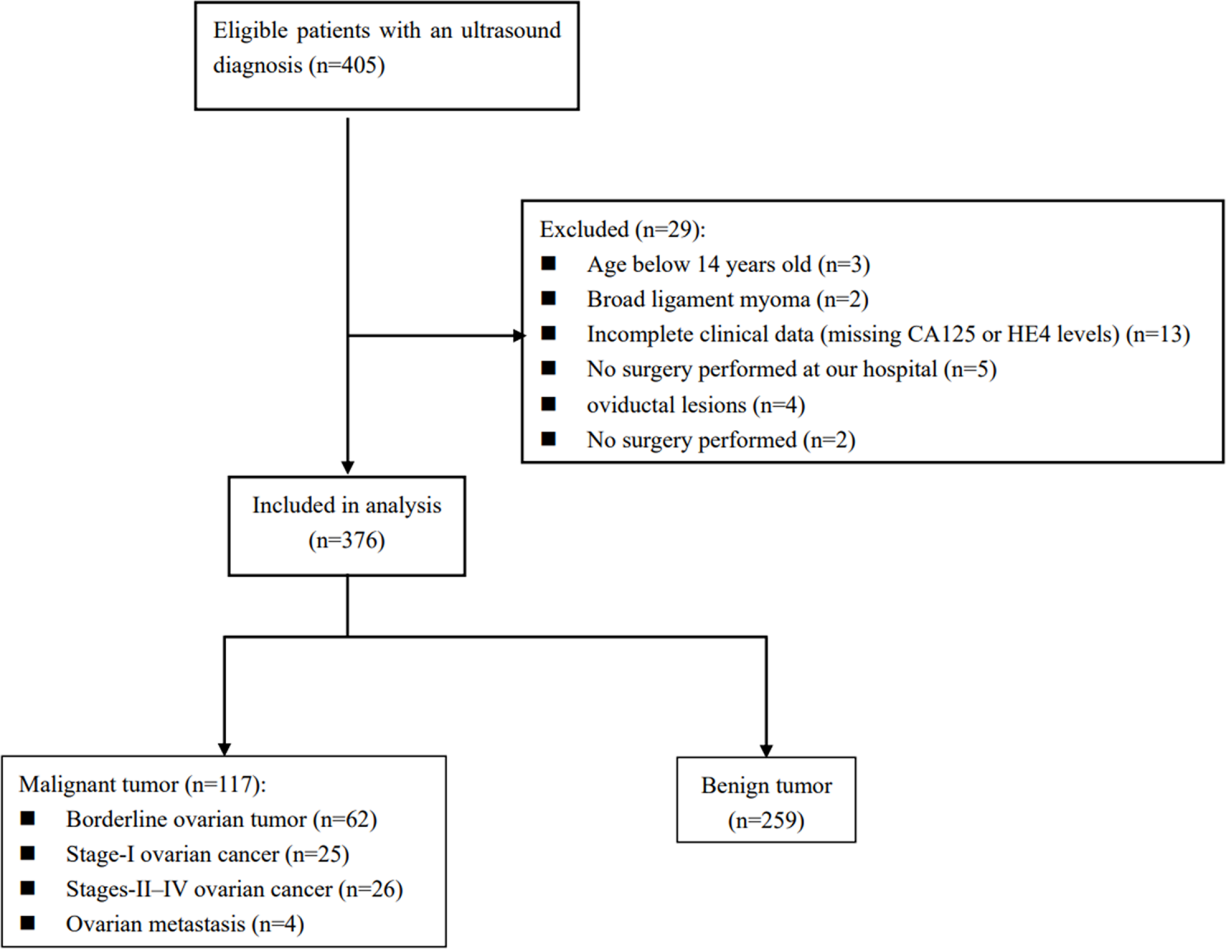
Diagram of how the study cohort was recruited from women diagnosed by ultrasound with adnexal masses based on the inclusion and exclusion criteria.

There were 405 patients with adnexal masses undergoing pre-operative ultrasound between August 2017 and September 2020. A total of 29 of these women were not included in this work because they were under 14 years old, the mass originated from the fallopian tube, insufficient clinical data, a broad ligament tumor on histology, failure to undergo surgery at our hospital, and they had not yet undergone surgery. Thus, 376 patients made up the final cohort (**Figure 1**).

Here is a listing of the histological results of the population studied in **Table 1**. Of these 376 women, 259 (68.9%) had benign ovarian tumors, whereas 117 (31.1%) had malignant ovarian tumors. There were 62 (16.5%) cases of BOT, 25 (6.6%) cases of Stage I OC, 26 (6.9%) cases of Stage II–IV OC, and 4 (1.1%) cases of metastatic ovary. Plasmacytoid cystadenoma and mucinous cystadenoma are the most commonly diagnosed benign tumors. On the other hand, clear cell carcinoma and serous high-grade carcinoma are the most common primary ovarian malignancies.

The clinical and ultrasonic characteristics of these women are summarized in **Table 2**. Those women with borderline tumors are younger than those with benign tumors, while their percentage in all malignant tumors is 52% (62/117). These findings showed that malignant tumors were slightly older than benign ones (*p* = 0.074). All patients were predominantly premenopausal (*p* = 0.000). Malignant tumors had a significantly higher maximum diameter, incidence of solid tissue, and incidence of papillary projections (*p* < 0.05 for all). More than triple the number of patients in the malignant group had more than 10 cyst locules (*p* = 0.005). A higher percentage of malignant patients had ascites than benign patients did (*p* = 0.031). We observed acoustic shadows only in one patient with malignant tumors.

**Table 2:** General clinical and ultrasonographic features of 376 ovarian benign and malignant tumors.

Characteristic	Benign (*n* = 259)	Malignant (*n* = 117)					*p*
		Borderline (*n* = 62)	OC Stage I (*n* = 25)	OC Stages II–IV (*n* = 26)	Ovarian metastasis (*n* = 4)	Total (*n* = 117)	
Age (years)	38.00	33.00	48.00	49.50	54.00	42.00	0.074^*^
	(27.00, 49.00)	(27.75, 42.25)	(38.50, 53.00)	(46.00, 59.00)	(50.50, 58.25)	(30.00, 50.00)
Menopausal status							0.000^+^
Premenopausal	216 (57.45)	56 (14.89)	17 (4.52)	16 (4.26)	1 (0.27)	90 (2.39)	
Postmenopausal	43 (11.44)	6 (1.60)	8 (2.13)	10 (2.66)	3 (0.80)	27 (7.18)	
Maximum diameter of lesion (mm)	76 (57, 102)	88 (59.50, 130.50)	117 (78, 146)	101 (76.50, 128.25)	84.5 (60, 107.50)	100 (66, 134)	0.000^*^
Solid tissue present	73 (19.41)	32 (8.50)	10 (2.66)	10 (2.66)	1 (0.27)	53 (14.10)	0.001^+^
Maximum diameter of largest solid component, if present (mm)	30 (14.50, 53.50)	63 (48.50, 93.00)	69 (38.75, 86.25)	34.50 (18.75, 54.25)	85 (75.00, 98.50)	52 (26.50, 75.00)	0.000^*^
Papillary projections present	47 (12.50)	40 (10.64)	8 (2.13)	6 (1.60)	0 (0)	54 (14.36)	0.000^+^
0	212 (56.38)	22 (5.85)	17 (4.52)	20 (5.32)	4 (1.06)	63 (16.76)	
1	27 (7.18)	16 (4.26)	1 (0.27)	1 (0.27)	0	18 (4.79)
2	9 (2.39)	6 (1.60)	0	0	0	6 (1.60)
3	3 (0.80)	5 (1.33)	2 (0.53)	2 (0.53)	0	9 (2.39)
>3	8 (2.13)	15 (3.99)	5 (1.33)	3 (0.80)	0	23 (6.12)
>10 cyst locules	11 (2.93)	10 (2.66)	4 (1.06)	0	0	14 (3.72)	0.005^+^
Acoustic shadows	23 (6.12)	0	1 (0.27)	0	0	1 (0.27)	0.002^++^
Ascites	13 (3.46)	7 (1.86)	1 (0.27)	5 (1.33)	0	13 (3.46)	0.031^+^

*For categorical data, n (%) is used, and for continuous data, the median (interquartile range) is used. The p-value for benign versus malignant groups is calculated with the following methods: *Mann–Whitney U test for continuous data, ^+^Chi-square test and ^++^Fisher exact test for categorical data. OC, ovarian cancer.

**Table 3** lists the results of analyzing serum CA125 and HE4 levels among different subtypes of ovarian tumors. For the CA125 level, the differences were statistically significant between benign and subtypes of malignant tumors except the ovarian metastasis (*p* < 0.05 for them), and between a Stage II–IV OC and an ovarian borderline tumor (*p* = 0.019). For the HE4 level, the differences were also statistically significant between the groups above except that between a benign tumor and an ovarian borderline tumor (*p* = 0.075). In addition, the differences in the HE4 level were also statistically significant between a Stage I OC and a Stage II–IV OC (*p* = 0.011), while no statistically significant differences were observed between an ovarian borderline tumor, a Stage I OC, and an ovarian metastasis tumor either in CA125 or in HE4 levels.

**Table 3:** Serum CA125 and HE4 level comparison between different subtypes of ovarian tumors.

Group	*n*	CA125		HE4
		Median		Median
		(Q1, Q3)		(Q1, Q3)
Benign	259	17.30		36.00
		(11.5, 29.0)		(30, 43)
Borderline	62	46.75		41.00
		(26.13, 120.68)		(32.75, 53.0)
OC Stage I	25	57.8		47.00
		(18.15, 265.60)		(32.00, 104.50)
OC Stages II–IV	26	362.0		217.00
		(93.23, 751.98)		(42.10, 682.00)
Ovarian metastasis	4	126.55		58.5
		(29.55, 275.3)		(51.5, 88.0)
*Z1*		−4.26		−3.221
*P1*		0.000		0.013
*Z2*		−6.441		−2.674
*P2*		0.000		0.075
*Z3*		−8.022		−7.727
*P3*		0.000		0.000
*Z4*		−2.424		−2.721
*P4*		0.154		0.065
*Z5*		−0.129		1.251
*P5*		1.000		1.000
*Z6*		3.099		5.186
*P6*		0.019		0.000
*Z7*		−0.578		−1.924
*P7*		1.000		0.543
*Z8*		−2.707		−3.267
*P8*		0.068		0.011
*Z9*		−0.611		−1.293
*P9*		1.000		1.000
*Z10*		0.799		0.407
*P10*		1.000		1.000

We present the data as a median (interquartile range); P1–P10 represent the comparison between different subtypes of ovarian tumors using Mann–Whitney U test and Z-statistic calculated. P1, benign vs. Stage I OC; P2, benign vs. borderline; P3, benign vs. Stage II–IV OC; P4, benign vs. ovarian metastasis; P5, borderline vs. Stage I OC; P6, borderline vs. Stage II–IV OC; P7, borderline vs. ovarian metastasis; P8, Stage I OC vs. Stage II–IV OC; P9, Stage I OC vs. ovarian metastasis; P10, Stage II–IV OC vs. ovarian metastasis.

### Assessing the differential diagnostic ability of the IOTA ADNEX model combined with HE4

**Figure 2:**
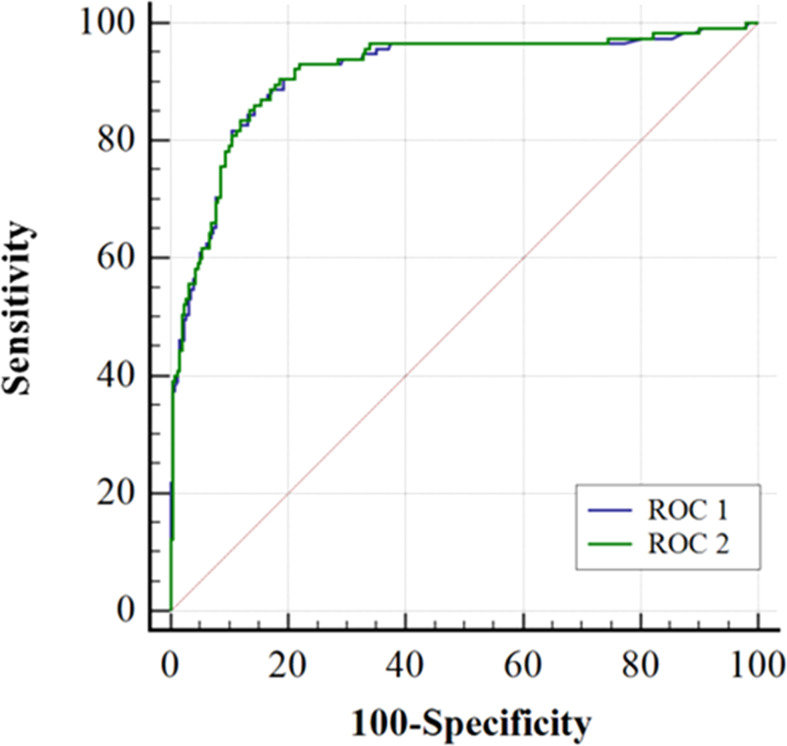
Receiver-operating characteristic curves (ROC) for the accuracy of ADNEX model alone (ROC 1) or in combination with HE4 (ROC 2) when separating malignant from benign ovarian tumors. The areas under ROC1 and ROC2 curves were 0.914 (0.881-0.941) and 0.916 (0.888-0.942), respectively. Comparing the AUC of the ROC 1 and the ROC 2 using DeLong’s test (P=0.0925).

**Figure 2** shows that the AUC of the ADNEX model alone or combined with HE4 in predicting benign tumors and malignant OCs was 0.914 (95% CI, 0.881–0.941) and 0.916 (95% CI, 0.883–0.942). The differences between them were not significant (*p* = 0.0925).

As the cutoff risk increases, the specificity gradually increases and the sensitivity gradually decreases simultaneously when the ADNEX model was used alone (**Table 4**). The sensitivity (0.87) and specificity (0.86) were balanced at the cutoff risk of 30.8%. The specificity was 0.81 when the ADNEX model was combined with HE4, which was higher than that when the cutoff risk of ADNEX model was 10% (0.73) or 15% (0.78).

**Table 4:** Efficacy of the IOTA ADNEX model alone or combined with HE4 to differentiate benign from malignant tumors.

ADNEX model	Cutoff	Sensitivity	Specificity	+LR	−LR	PPV	NPV
Benign vs. malignant	5%	0.97 (0.91–0.99)	0.55 (0.49–0.61)	2.16 (1.90–2.50)	0.06 (0.02–0.20)	0.49 (0.45–0.52)	0.97 (0.93–0.99)
	10%	0.93 (0.87–0.97)	0.73 (0.67–0.78)	3.39 (2.80–4.20)	0.10 (0.05–0.20)	0.60 (0.55–0.65)	0.96 (0.92–0.98)
	15%	0.93 (0.87–0.97)	0.78 (0.72–0.83)	4.15 (3.30–5.20)	0.09 (0.05–0.20)	0.65 (0.05–0.20)	0.96 (0.93–0.98)
	30.8%	0.87 (0.78–0.92)	0.86 (0.81–0.90)	6.03 (4.40–8.20)	0.16 (0.10–0.30)	0.73 (0.66–0.78)	0.93 (0.90–0.96)
Benign vs. malignant combining with HE4	0.171	0.90 (0.84–0.95)	0.81 (0.76–0.86)	4.88 (3.80–6.30)	0.12 (0.07–0.20)	0.68 (62.50–73.80)	0.95 (91.60–97.10)

The likelihood ratios are +LR and –LR. The predictive values are NPV and PPV.

Results of the IOTA ADNEX model alone or combined with HE4 for discriminating between different subclassifications of ovarian tumors are listed in **Table 5**. Their performance in discriminating benign from the different subtypes of malignant tumors is excellent. The AUCs vary between 0.860 and 0.975 when the ADNEX model was combined with HE4 and between 0.841 and 0.977 when the ADNEX model was used alone. The difference between the groups above was not statistically significant (*p* > 0.05 for all). For the differential diagnosis ability between subclassifications of malignant tumors, the AUCs vary between 0.697 and 0.838 when ADNEX was used alone and increased to between 0.760 and 0.903 when the ADNEX model was combined with HE4. The difference in the differential diagnostic ability between an ovarian borderline tumor and a Stage II–IV OC was statistically significant (*p* = 0.0257). However, both of them have poor differential diagnostics for a Stage I OC and an ovarian metastasis, with an AUC of 0.71 and 0.76, respectively.

**Table 5:** Comparison of the differential diagnostic ability of the IOTA ADNEX model alone or combined with HE4 in the identification of various types of ovarian tumors.

Discrimination	AUC (95% CI)		*p*
	ADNEX model combined with HE4	ADNEX model
Benign vs. malignant	0.916 (0.883–0.942)	0.914 (0.881–0.941)	0.0925
Benign vs. BOT	0.860 (0.817–0.896)	0.841 (0.796–0.879)	0.2885
Benign vs. Stage I OC	0.955 (0.924–0.976)	0.948 (0.915–0.971)	0.2183
Benign vs. Stage II–IV OC	0.975 (0.949–0.990)	0.977 (0.952–0.991)	0.6051
Benign vs. metastasis	0.933 (0.896–0.960)	0.937 (0.901–0.963)	0.3517
BOT vs. Stage I OC	0.813 (0.714–0.890)	0.758 (0.653–0.844)	0.1200
BOT vs. Stage II–IV OC	0.903 (0.820–0.956)	0.838 (0.743–0.909)	0.0257
BOT vs. metastasis	0.821 (0.705–0.905)	0.773 (0.651–0.868)	0.3336
Stage I OC vs. Stage II–IV OC	0.812 (0.678–0.908)	0.734 (0.592–0.848)	0.0823
Stage I OC vs. metastasis	0.760 (0.566–0.898)	0.710 (0.513–0.862)	0.1587
Stage II–IV OC vs. metastasis	0.885 (0.715–0.972)	0.697 (0.503–0.850)	0.2960

The area under receiver operating characteristic curve (AUC) of the IOTA ADNEX model alone or in combination with HE4 is compared using DeLong’s test. BOT is borderline ovarian tumor; OC is ovarian cancer.

Regarding the differential diagnosis of an ovarian borderline tumor and a Stage II–IV OC, the AUC from 0.838 increased to 0.903 after the ADNEX model in combination with HE4, and the sensitivity increased from 0.73 to 0.85, while the specificity was maintained (**Table 6**).

**Table 6:** An assessment of the IOTA ADNEX model combined with HE4 to differentiate an ovarian borderline tumor from a Stage II–IV OC.

ADNEX model	Sensitivity	Specificity	+LR	−LR	PPV	NPV	Cutoff	Youden index	*p*
Borderline vs. Stage II–IV OC	0.73 (0.52, 0.88)	0.90 (0.80, 0.96)	7.31 (3.3, 16.2)	0.3 (0.2, 0.6)	0.76 (0.60, 0.88)	0.89 (0.80, 0.94)	31.4	0.631	0.0257
Borderline vs. Stage II–IV OC combined with HE4	0.85 (0.65, 0.96)	0.90 (0.80, 0.96)	8.46 (3.9, 18.4)	0.17 (0.07, 0.4)	0.79 (0.63, 0.89)	0.93 (0.85, 0.97)	0.195	0.746	

NPV represents the negative predictive value; PPV represents the positive predictive value. The likelihood ratios are positive (+LR) or negative (–LR).

## Discussion

A correct and early diagnosis of OC can significantly increase the patient’s chances of survival (27, 28). As previously reported, the main models and scoring systems for diagnosing ovarian tumors were compared and analyzed. When diagnosing benign and malignant ovarian tumors, the ADNEX model had a higher AUC value and sensitivity (0.94 and 96.5%) than the risk of malignancy index (RMI) (0.85 and 89%), the risk of ovarian malignancy algorithm (ROMA) (0.84 and 91%), and the Copenhagen index (CPH-I) (0.81 and 69%) (29–32). The diagnostic performance of RMI and CPH-I is affected by the base rate of OC (33). The main limitations of the RMI are its lack of an estimated risk of malignancy, and its high reliance on serum CA125, which makes it relatively insensitive to borderline and early-stage invasive diseases, especially in women who are premenopausal (6, 34). In addition, a multicenter cohort study comparing six prediction models (RMI, LR2, Simple Rules, Simple Rules risk model, and the ADNEX model with or without CA125), conducted in 17 centers, demonstrated that the IOTA ADNEX model and the IOTA Simple Rules risk model were the best (6, 35). Furthermore, the ADNEX model is capable of classifying subtypes of ovarian malignancies. Therefore, the ADNEX model is currently the most research-valued diagnostic model.

At our Gynecological Oncology Center, the IOTA ADNEX model is effective at differentiating benign from malignant ovarian tumors, which is in line with the research of domestic and foreign scholars (35). As far as the IOTA ADNEX cutoff value is concerned, 10% was usually selected as the cutoff point in current studies, although the sensitivity (varying from 93.3% to 98%) is high and specificity is low (varying from 62% to 77.8%). Therefore, some scholars have proposed to use 15% as the cutoff point to ensure that its sensitivity is >90%, and its specificity can be increased (varying from 72.7% to 83.7%) (15, 29, 36–39). In our study, when the IOTA ADNEX model was combined with HE4, the sensitivity of differentiating benign from malignant ovarian tumors was 90.43%, and the specificity could be increased to 81.47%. It can reduce the number of false positives, optimize resource allocation, and reduce treatment cost due to its high specificity.

Currently, ultrasound-based predictive models for the preoperative correct detection of an ovarian borderline tumor, a Stage I OC, and a metastatic tumor remain a challenge (40, 41). Previous studies have shown that almost one-half of all borderline tumors are incorrectly diagnosed or classified by subjective ultrasound evaluation, and diagnostic problems associated with difficult borderline tumors cannot be solved by logistic regression models (42). In comparison to the IOTA ADNEX model, the simple rules of IOTA and non-IOTA models perform poorly when it comes to identifying BOTs and Stage I OCs (43, 44). Consistent with previous studies (44, 45), the IOTA ADNEX performed excellently in terms of detecting most types of adnexal masses in this work (an AUC of 0.697 to 0.977 was observed). Nevertheless, the model performed poorly at distinguishing between an ovarian borderline tumor and a Stage I OC (AUC, 0.758), between an ovarian borderline and a metastatic tumor (AUC, 0.773), between a Stage I OC and a metastatic tumor (AUC, 0.710), between a Stage I OC and a Stage II–IV OC (AUC, 0.734), and between a Stage II–IV OC and a metastatic tumor (AUC, 0.697). The results were similar to or lower than previous studies (29, 37).

In order to enhance early OC detection, we combined the IOTA ADNEX model with HE4 for the first time. Serum CA125 was a clinical indicator in the ADNEX model that may be impacted by infections and pregnancy, having a lower sensitivity and a high false-positive rate (30, 46–49). Serum HE4, an important supplementary indicator of CA125, had a similar sensitivity and a higher specificity, especially for asymptomatic patients with Stage I OC, and had been recommended as a potential biomarker (50). Our results showed that the ADNEX model, whether combined with HE4 or not, was excellent for the differential diagnosis of benign and malignant ovarian tumors (AUC of 0.916 and 0.914, respectively). The specificity of the combined diagnosis of the ADNEX model and HE4 is greater than that of the 10% cutoff risk in the ADNEX model, while the sensitivities are both greater than 90%. The differential diagnosis ability improved after the ADNEX model was combined with HE4 compared with the ADNEX model used alone in distinguishing between most of the different types of ovarian malignancies, with the AUC varying between 0.697 and 0.838 and increased to between 0.760 and 0.903. However, it was still ineffective at distinguishing between Stage I OC and metastasis tumor (AUC of 0.760 and 0.710, respectively). Most of the differences above were not statistically significant (*p* > 0.05 for them) except for an ovarian borderline tumor vs. a Stage II–IV OC (*p* = 0.0257), which may be related to the non-obvious expression of serum CA125 and HE4 levels that was significantly different between borderline, Stage I OC, and metastatic OC groups (as shown in **Table 3**) or may be related to the limited number of cases in our study. Therefore, further research should be conducted for biomarkers targeting early diagnosis of OC. Some researchers have proposed ovarian tumor stem cell-specific biomarkers such as CA24, CD44, CD133, and SSEA, and others have proposed the unique peritoneal microbial profile of OC patients. Perhaps, these biomarkers have important biological and clinical significance in terms of the early detection rate of OC (51, 52).

Our study has shortcomings. First, this work was conducted in one hospital, with limited data collection. Second, the feasibility was not verified either in internal or in external gynecological oncology centers with new data. We will gradually overcome these problems in a follow-up research.

In conclusion, the ADNEX model, alone or combined with HE4, performs excellently to determine the benignity or malignancy of an ovarian tumor, while the specificity was higher when combined with HE4. The ADNEX model combined with HE4 can improve the differential diagnosis ability and the sensitivity of an ovarian borderline tumor and a Stage II–IV OC.

## Data availability statement

The original contributions presented in the study are included in the article/supplementary material. Further inquiries can be directed to the corresponding author.

## Ethics statement

The studies involving human participants were reviewed and approved by Institutional Ethics Committee of Women and Children’s Hospital of Chongqing Medical University. Written informed consent to participate in this study was provided by the participants’ legal guardian/next of kin. Written informed consent was obtained from the individual(s), and minor(s)’ legal guardian/next of kin, for the publication of any potentially identifiable images or data included in this article.

## Author contributions

JT: project development. JT and SY: data collection, data analysis, and manuscript writing. YR, MW, JL, CC, and CW: data collection and manuscript writing. All authors contributed to the article and approved the submitted version.

## Funding

The study was supported by the Technological Innovation and Application Development Project of Chongqing (cstc2019jscx-msxmX0235).

## Conflict of interest

The authors declare that the research was conducted in the absence of any commercial or financial relationships that could be construed as a potential conflict of interest.

## Publisher’s note

All claims expressed in this article are solely those of the authors and do not necessarily represent those of their affiliated organizations, or those of the publisher, the editors and the reviewers. Any product that may be evaluated in this article, or claim that may be made by its manufacturer, is not guaranteed or endorsed by the publisher.

## References

[1] Bray F Ferlay J Soerjomataram I Siegel RL Torre LA Jemal A. Global cancer statistics 2018: GLOBOCAN estimates of incidence and mortality worldwide for 36 cancers in 185 countries. CA Cancer J Clin (2018) 68(6):394–424. doi: 10.3322/caac.21492 30207593

[2] Sung H Ferlay J Siegel RL Laversanne M Soerjomataram I Jemal A. Global cancer statistics 2020: GLOBOCAN estimates of incidence and mortality worldwide for 36 cancers in 185 countries. CA Cancer J Clin (2021) 71(3):209–49. doi: 10.3322/caac.21660 33538338

[3] Stewart C Ralyea C Lockwood S. Ovarian cancer: An integrated review. Semin Oncol Nurs (2019) 35(2):151–6. doi: 10.1016/j.soncn.2019.02.001 30867104

[4] Webb PM Jordan SJ. Epidemiology of epithelial ovarian cancer. Best Pract Res Clin Obstet Gynaecol (2017) 41:3–14. doi: 10.1016/j.bpobgyn.2016.08.006 27743768

[5] Cancer stat facts: Ovarian cancer (2020). Available at: https://seer.cancer.gov/statfacts/html/ovary.html.

[6] Timmerman D Planchamp F Bourne T Landolfo C du Bois A Chiva L. ESGO/ISUOG/IOTA/ESGE consensus statement on preoperative diagnosis of ovarian tumors. Ultrasound Obstet Gynecol (2021) 58(1):148–68. doi: 10.1002/uog.23635 33794043

[7] Timmerman D Testa AC Bourne T Ferrazzi E Ameye L Konstantinovic ML. Logistic regression model to distinguish between the benign and malignant adnexal mass before surgery: a multicenter study by the international ovarian tumor analysis group. J Clin Oncol (2005) 23(34):8794–801. doi: 10.1200/JCO.2005.01.7632 16314639

[8] Timmerman D Van Calster B Testa A Savelli L Fischerova D Froyman W. Predicting the risk of malignancy in adnexal masses based on the simple rules from the international ovarian tumor analysis group. Am J Obstet Gynecol (2016) 214(4):424–37. doi: 10.1016/j.ajog.2016.01.007 26800772

[9] Calster BV Hoorde KV Froyman W Kaijser J Wynants L Landoldo C. Practical guidance for applying the ADNEX model from the IOTA group to discriminate between different subtypes of adnexal tumors. Facts Views Vis Obgyn (2015) 7(1):32–41. 25897370 PMC4402441

[10] Qian L Du Q Jiang M Yuan F Chen H Feng W. Comparison of the diagnostic performances of ultrasound-based models for predicting malignancy in patients with adnexal masses. Front Oncol (2021) 11:673722. doi: 10.3389/fonc.2021.673722 34141619 PMC8204044

[11] He P Wang JJ Duan W Song C Yang Y Wu QQ. Estimating the risk of malignancy of adnexal masses: validation of the ADNEX model in the hands of nonexpert ultrasonographers in a gynaecological oncology centre in China. J Ovarian Res (2021) 14(1):169. doi: 10.1186/s13048-021-00922-w 34857005 PMC8638097

[12] Peng XS Ma Y Wang LL Li HX Zheng XL Liu Y. Evaluation of the diagnostic value of the ultrasound ADNEX model for benign and malignant ovarian tumors. Int J Gen Med (2021) 14:5665–73. doi: 10.2147/IJGM.S328010 PMC8454417 34557021

[13] Tug N Yassa M Akif Sargin M Dogan Taymur B Sandal K Ertnuc M. Preoperative discriminating performance of the IOTA-ADNEX model and comparison with risk of malignancy index: an external validation in a non-gynecologic oncology tertiary center. Eur J Gynaecol Oncol (2020) 41(2)::200–7. doi: 10.31083/j.ejgo.2020.02.4971

[14] Nohuz E De Simone L Chene G. Reliability of IOTA score and ADNEX model in the screening of ovarian malignancy in postmenopausal women. J Gynecol Obstet Hum Reprod (2019) 48(2):103–7. doi: 10.1016/j.jogoh.2018.04.012 29709594

[15] Meys EMJ Jeelof LS Achten NMJ Slangen BFM Lambrechts S Kruitwagen R. Estimating risk of malignancy in adnexal masses: external validation of the ADNEX model and comparison with other frequently used ultrasound methods. Ultrasound Obstet Gynecol (2017) 49(6):784–92. doi: 10.1002/uog.17225 PMC5488216 27514486

[16] Joyeux E Miras T Masquin I Duglet PE Astruc K Douvier S. Before surgery predictability of malignant ovarian tumors based on ADNEX model and its use in clinical practice. Gynecol Obstet Fertil (2016) 44(10):557–64. doi: 10.1016/j.gyobfe.2016.07.007 27568408

[17] Capriglione S Luvero D Plotti F Terranova C Montera R Scaletta G. Ovarian cancer recurrence and early detection: may HE4 play a key role in this open challenge? a systematic review of literature. Med Oncol (2017) 34(9):164. doi: 10.1007/s12032-017-1026-y 28825178

[18] Moore RG Jabre-Raughley M Brown AK Robison KM Miller MC Allard WJ. Comparison of a novel multiple marker assay vs the risk of malignancy index for the prediction of epithelial ovarian cancer in patients with a pelvic mass. Am J Obstet Gynecol (2010) 203(3):228.e1–6. doi: 10.1016/j.ajog.2010.03.043 PMC3594101 20471625

[19] Lin J Qin J Sangvatanakul V. Human epididymis protein 4 for differential diagnosis between benign gynecologic disease and ovarian cancer: a systematic review and meta-analysis. Eur J Obstet Gynecol Reprod Biol (2013) 167(1):81–5. doi: 10.1016/j.ejogrb.2012.10.036 23228410

[20] Moore RG Brown AK Miller MC Skates S Allard WJ Verch T. The use of multiple novel tumor biomarkers for the detection of ovarian carcinoma in patients with a pelvic mass. Gynecol Oncol (2008) 108(2):402–8. doi: 10.1016/j.ygyno.2007.10.017 18061248

[21] Melo A Verissimo R Farinha M Martins NN Martins FN. Discriminative value of CA-125, HE4, risk of (RMI-II) and risk of malignancy algorithm (ROMA) in the differential diagnosis of pelvic masses: conclusions from a referral centre in Portugal. J Obstet Gynaecol (2018) 38(8):1140–5. doi: 10.1080/01443615.2018.1457632 29884096

[22] Molina R Escudero JM Auge JM Filella X Foj L Torne A. HE4 a novel tumour marker for ovarian cancer: comparison with CA 125 and ROMA algorithm in patients with gynaecological diseases. Tumour Biol (2011) 32(6):1087–95. doi: 10.1007/s13277-011-0204-3 PMC3195682 21863264

[23] Scaletta G Plotti F Luvero D Capriglione S Montera R Miranda A. The role of novel biomarker HE4 in the diagnosis, prognosis and follow-up of ovarian cancer: a systematic review. Expert Rev Anticancer Ther (2017) 17(9):827–39. doi: 10.1080/14737140.2017.1360138 28756722

[24] Timmerman D Valentin L Bourne TH Collins WP Verrelst H Vergote I. Terms, definitions and measurements to describe the sonographic features of adnexal tumors: a consensus opinion from the international ovarian tumor analysis (IOTA) grop. Ultrasound Obstet Gynecol (2000) 16:500–5. 10.1046/j.1469-0705.2000.00287.x 11169340

[25] Meinhold-Heerlein I Fotopoulou C Harter P Kurzeder C Mustea A Wimberger P. The new WHO classification of ovarian, fallopian tube, and primary peritoneal cancer and its clinical implications. Arch Gynecol Obstet (2016) 293(4):695–700. doi: 10.1007/s00404-016-4035-8 26894303

[26] Prat J Oncology FCoG. Staging classification for cancer of the ovary, fallopian tube, and peritoneum. . Int J Gynaecol Obstet (2014) 124(1):1–5. doi: 10.1016/j.ijgo.2013.10.001 24219974

[27] Jacobs IJ Menon U Ryan A Gentry-Maharaj A Burnell M Kalsi JK. Ovarian cancer screening and mortality in the UK collaborative trial of ovarian cancer screening (UKCTOCS): a randomised controlled trial. Lancet (2016) 387(10022):945–56. doi: 10.1016/s0140-6736(15)01224-6 PMC4779792 26707054

[28] Huang Y Ming X Li B Li Z. Histological characteristics and early-stage diagnosis are associated with better survival in young patients with epithelial ovarian cancer: A retrospective analysis based on surveillance epidemiology and end results database. Front Oncol (2020) 10:595789. doi: 10.3389/fonc.2020.595789 33425749 PMC7787102

[29] Van Calster B Van Hoorde K Valentin L Testa AC Fischerova D Van Holsbeke C. Evaluating the risk of ovarian cancer before surgery using the ADNEX model to differentiate between benign, borderline, early and advanced stage invasive, and secondary metastatic tumours: prospective multicentre diagnostic study. BMJ (2014) 349:g5920. doi: 10.1136/bmj.g5920 25320247 PMC4198550

[30] Lycke M Kristjansdottir B Sundfeldt K. A multicenter clinical trial validating the performance of HE4, CA125, risk of ovarian malignancy algorithm and risk of malignancy index. Gynecol Oncol (2018) 151(1):159–65. doi: 10.1016/j.ygyno.2018.08.025 30149898

[31] Minar L Felsinger M Cermakova Z Zlamal F Bienertova-Vasku J. Comparison of the Copenhagen index versus ROMA for the preoperative assessment of women with ovarian tumors. Int J Gynaecol Obstet (2018) 140(2):241–6. doi: 10.1002/ijgo.12371 29086914

[32] Poonyakanok V Tanmahasamut P Jaishuen A Wongwananuruk T Asumpinwong C Panichyawat N. Preoperative evaluation of the ADNEX model for the prediction of the ovarian cancer risk of adnexal masses at siriraj hospital. Gynecol Obstet Invest (2021) 86(1-2):132–8. doi: 10.1159/000513517 33596584

[33] Rolfsen ALD Dahl AA Pripp AH Dorum A. Base rate of ovarian cancer on algorithms in patients with a pelvic mass. Int J Gynecol Cancer (2020) 30(11):1775–9. doi: 10.1136/ijgc-2020-001416 PMC7656145 32699016

[34] Timmerman D Van Calster B Jurkovic D Valentin L Testa AC Bernard JP. Inclusion of CA-125 does not improve mathematical models developed to distinguish between benign and malignant adnexal tumors. J Clin Oncol (2007) 25(27):4194–200. doi: 10.1200/JCO.2006.09.5943 17698805

[35] Van Calster B Valentin L Froyman W Landolfo C Ceusters J Testa AC. Validation of models to diagnose ovarian cancer in patients managed surgically or conservatively: multicentre cohort study. BMJ (2020) 370:m2614. doi: 10.1136/bmj.m2614 32732303 PMC7391073

[36] Westwood M Ramaekers B Lang S Grimm S Deshpande S de Kock S. Risk scores to guide referral decisions for people with suspected ovarian cancer in secondary care: a systematic review and cost-effectiveness analysis. Health Technol Assess (2018) 22(44):1–264. doi: 10.3310/hta22440 PMC6139475 30165935

[37] Chen H Qian L Jiang M Du Q Yuan F Feng W. Performance of IOTA ADNEX model in evaluating adnexal masses in a gynecological oncology center in China. Ultrasound Obstet Gynecol (2019) 54(6):815–22. doi: 10.1002/uog.20363 31152572

[38] Huang X Wang Z Zhang M Luo H. Diagnostic accuracy of the ADNEX model for ovarian cancer at the 15% cut-off value: A systematic review and meta-analysis. Front Oncol (2021) 11:684257. doi: 10.3389/fonc.2021.684257 34222006 PMC8247918

[39] Hiett AK Sonek JD Guy M Reid TJ. Performance of IOTA simple rules, simple rules risk assessment, ADNEX model and O-RADS in differentiating between benign and malignant adnexal lesions in north American women. Ultrasound Obstet Gynecol (2022) 59(5):668–76. doi: 10.1002/uog.24777 34533862

[40] Campos C Sarian LO Jales RM Hartman C Araujo KG Pitta D. Performance of the risk of malignancy index for discriminating malignant tumors in women with adnexal masses. J Ultrasound Med (2016) 35(1):143–52. doi: 10.7863/ultra.15.01068 26657746

[41] Van Holsbeke C Van Calster B Bourne T Ajossa S Testa AC Guerriero S. External validation of diagnostic models to estimate the risk of malignancy in adnexal masses. Clin Cancer Res (2012) 18(3):815–25. doi: 10.1158/1078-0432.CCR-11-0879 22114135

[42] Valentin L Ameye L Jurkovic D Metzger U Lecuru F Van Huffel S. Which extrauterine pelvic masses are difficult to correctly classify as benign or malignant on the basis of ultrasound findings and is there a way of making a correct diagnosis? Ultrasound Obstet Gynecol (2006) 27(4):438–44. doi: 10.1002/uog.2707 16526098

[43] Timmerman D Testa AC Bourne T Ameye L Jurkovic D Van Holsbeke C. Simple ultrasound-based rules for the diagnosis of ovarian cancer. Ultrasound Obstet Gynecol (2008) 31(6):681–90. doi: 10.1002/uog.5365 18504770

[44] Araujo KG Jales RM Pereira PN Yoshida A de Angelo Andrade L Sarian LO. Performance of the IOTA ADNEX model in preoperative discrimination of adnexal masses in a gynecological oncology center. Ultrasound Obstet Gynecol (2017) 49(6):778–83. doi: 10.1002/uog.15963 27194129

[45] Sayasneh A Ferrara L De Cock B Saso S Al-Memar M Johnson S. Evaluating the risk of ovarian cancer before surgery using the ADNEX model: a multicentre external validation study. Br J Cancer (2016) 115(5):542–8. doi: 10.1038/bjc.2016.227 PMC4997550 27482647

[46] Soletormos G Duffy MJ Othman Abu Hassan S Verheijen RH Tholander B Bast RC Jr.. Clinical use of cancer biomarkers in epithelial ovarian cancer: Updated guidelines from the European group on tumor markers. Int J Gynecol Cancer (2016) 26(1):43–51. doi: 10.1097/IGC.0000000000000586 26588231 PMC4679342

[47] Cramer DW Vitonis AF Welch WR Terry KL Goodman A Rueda BR. Correlates of the preoperative level of CA125 at presentation of ovarian cancer. Gynecol Oncol (2010) 119(3):462–8. doi: 10.1016/j.ygyno.2010.08.028 PMC2980911 20850174

[48] Babic A Cramer DW Kelemen LE Kobel M Steed H Webb PM. Predictors of pretreatment CA125 at ovarian cancer diagnosis: a pooled analysis in the ovarian cancer association consortium. Cancer Causes Control (2017) 28(5):459–68. doi: 10.1007/s10552-016-0841-3 PMC5593071 28050675

[49] Johnson CC Kessel B Riley TL Ragard LR Williams CR Xu JL. The epidemiology of CA-125 in women without evidence of ovarian cancer in the prostate, lung, colorectal and ovarian cancer (PLCO) screening trial. Gynecol Oncol (2008) 110(3):383–9. doi: 10.1016/j.ygyno.2008.05.006 PMC3744195 18586313

[50] Cao H You D Lan Z Ye H Hou M Xi M. Prognostic value of serum and tissue HE4 expression in ovarian cancer: a systematic review with meta-analysis of 90 studies. Expert Rev Mol Diagn (2018) 18(4):371–83. doi: 10.1080/14737159.2018.1457436 29569984

[51] Miao R Badger TC Groesch K Diaz-Sylvester PL Wilson T Ghareeb A. Assessment of peritoneal microbial features and tumor marker levels as potential diagnostic tools for ovarian cancer. PloS One (2020) 15(1):e0227707. doi: 10.1371/journal.pone.0227707 31917801 PMC6952086

[52] Muinao T Deka Boruah HP Pal M. Diagnostic and prognostic biomarkers in ovarian cancer and the potential roles of cancer stem cells - an updated review. Exp Cell Res (2018) 362(1):1–10. doi: 10.1016/j.yexcr.2017.10.018 29079264

